# Part-time work and sickness absence – an organization-level analysis

**DOI:** 10.1186/s12889-023-17189-z

**Published:** 2023-11-14

**Authors:** Dag Ingvar Jacobsen, Viktoria Fjelde

**Affiliations:** University of Agder, Servicebox 422, Kristiansand, 4604 Norway

**Keywords:** Part-time work, Sickness absence, Human Resource Management architecture, Working hours

## Abstract

**Background:**

Absenteeism is consistently higher in public than in private organizations, as is the use of part-time employment. The aim of this study is to identify whether there is a relationship between part-time work and sickness absence at the organizational level.

**Methods:**

The data is a six-year panel for the full population of Norwegian municipalities (N = 422), linking objective register data on both part-time employment and sickness absence. Using OLS regression with fixed effects for municipality and time, we estimate the statistical effects of the municipalities’ use of part-time work on sickness absence.

**Results:**

The bivariate correlation between percentage position at the municipal level and percentage sickness absence is positive and significant (Pearson’s r = .25, sig LE 0.01). When controlling for fixed effects for municipality and time, as well as municipality economy, municipality size, ratio of female employees in the municipality and characteristics of the general population, the multivariate regression coefficient is still positive but insignificant (coefficient = 1.56, robust standard error = 1.31).

**Conclusions:**

The main findings are that the organizations’ use of part-time work is unrelated to sickness absence indicating that organizations with extensive use of part-time work do not experience higher levels of absenteeism than those having less extensive use of part-time employees.

## Background

Absence from work – or absenteeism – is a serious problem for all organizations, especially public organizations as absenteeism is much higher in the public than in the private sector, a difference that cuts across national borders and is stable over time [1]. Research attempting to explain variations in sickness absence has focused on individual characteristics like gender, age, education, personal health and lifestyle [2–5] and family related conditions [6, 7], organizational factors like organization size [8], job design [9, 10], balance between job demands and resources [11–13], temporality of work (shift work) [14, 15], leadership [16, 17], and job autonomy [18, 19], and sector and business employment [1, 20, 21], national differences including institutional arrangements for registering and compensating absence from work [21–25].

Part-time work is a common, and in many countries increasingly so, phenomenon. In 2020 around 17% of the US workforce worked part-time, while the average in the 27 EU countries was almost 15% in 2021 [26]. Research on eventual links between part-time work and absenteeism is, however, rather scarce. This is somewhat surprising given that part-time work, together with overtime and shift work, is classified by the OECD as “non-standard work”, claiming that employees in this type of work are “(…) worse off in many aspects of job quality, such as earnings, job security or access to training.” [27]. Furthermore, organizations vary widely on their use of part-time work [28–30] and is by some regarded as an important part of any organization’s “Human Resource Management architecture” [31]. Still, two scoping reviews covering 40 years of research on causes for absenteeism [32, 33] do not mention working hours as a possible antecedent.

Moreover, the findings from empirical studies are “highly inconclusive” [34]. Most studies report part-time employment (PTE) to be associated with higher absenteeism [35–38], while somewhat fewer report no significant association between the two [39–41]. A few studies conclude that PTE is associated with lower absenteeism [42, 43]. One possible reason for these inconclusive results is that research is almost exclusively conducted at the individual level. As part-time work may have both beneficial effects for some (flexible work, lower workload, etc.), and detrimental effects for others (less integrated into the workplace, lower pay, etc.), the aggregated effects may level each other out. This study circumvents this problem by studying the relationship between part-time work and sickness absence at the organizational or aggregated level, thus measuring the *net organizational effect* of part-time work on sickness absence.

To begin with, we outline two competing hypotheses regarding part-time work and absenteeism. These hypotheses are then tested using data from Norwegian municipalities. Objective register data on part-time work and absenteeism is aggregated to the organizational (municipal) level. Thus, the study empirically investigates whether organizational differences across municipalities in the use of part-time is associated with organizational differences in absenteeism. The empirical data is composed of almost all Norwegian municipalities (N = 422) in the period between 2014 and 2019 (panel data). The analysis is conducted using ordinary least square (OLS) linear regression with municipality and time fixed effects to account for unobserved differences between municipalities and years.

## Theory and hypotheses

Although working part-time may or may not be an individual decision (one cannot find a full-time job), staying away from work will always be an individual decision. This decision may be voluntary in the sense that one is able to go to work but decides to stay at home or do something else. And it may be involuntary in the sense that one wants to go to work, but it is impossible because of sickness or other factors hindering the person [44, 45].

Thus, explaining sickness absence at the municipal or organizational level will have to rely on micro-processes taking place at the individual level [46]. Sickness absence at the organizational level is then the sum of the individual sickness absence, or the aggregate of the individual decisions to go to work or not. An eventual link between sickness absence and part-time work at the organizational level must be explained by mechanisms functioning at the individual level. Several speculations, based in different theories of human behavior, have been put forward to explain possible links between part-time work and absenteeism. Two different streams of theory and research can be detected: one that argues that part-time will reduce absenteeism and the opposite, that part-time will increase absenteeism.

Starting with arguments that part-time work will decrease absenteeism, one is that organizations offering part-time work can be regarded as providing flexible work schedules [47], making it easier for employees to strike the right balance between work and family/leisure. Studies based in theories on work-family balance [48], assume that working part-time is a way to improve the balance between demands from home and from work [49–54]. Organizations with a higher level of part-time workers may thus represent a working environment with greater possibilities for easing the potential conflict between work and home and should thus result in reduced absenteeism. The above argument assumes that employees freely can choose how many hours they will work (voluntary part-time). In many instances, this will not be the case [55]. Rather, employees will often have no other choice, but the number of hours offered by the organization. This may result in a situation where employees end up working fewer hours than wanted (involuntary part-time). As noted by Jacobsen & Fjeldbraaten [56], this may set employees in a situation where they “(…) will be more hesitant to take some days off as this might impair future possibilities of getting a higher percentage of work.” Working less than wanted may thus have a disciplining effect on employees, decreasing their absenteeism.

Part-time work may also reduce the physical and psychic strain for many employees. Previous research has indicated quite clearly that probabilities for developing both physical and mental problems increase with the number of hours worked [57, 58], and that a reduction in working hours seems to reduce both physical and mental problems for employees [59]. Although most of these studies focus on working longer hours than “normal”, they may also be relevant for employees working less than “normal”. Finally, there may be just a statistical artefact explaining why part-time workers may have lower absenteeism than full-time workers. Part-time workers will usually also work fewer days during a week or month. Thus, there will also be a higher probability for these employees that sickness will strike them on days that they are not expected to work. The more days one is working, the higher the statistical probability of being sick on one of the working days.

Based on the above arguments we propose the following hypothesis:

H1: Absence at the organizational level will decrease with higher levels of part-time work in the organization.

Moving to the arguments that part-time will increase absenteeism, the most common seems to be connected to a lack of integration in the organization [11, 12, 56, 60]. Most studies following this line of reasoning are based in theories related to partial inclusion [61, 62] and organizational commitment [63, 64], especially affective organizational commitment (AOC) [65, 66]. As AOC usually is a result of organizational socialization, working less than normal will almost automatically expose the employee less to the organizational elements central in the socialization process: colleagues, management, and clientele. Thus, part-time work may entail lower affective commitment. And, as several studies have shown that high AOC is linked to low absenteeism [12, 67, 68], we will expect that organizations with more part-time workers will exhibit higher levels of sickness absence.

A second effect may come through an attraction/selection process where employees with higher propensity for absenteeism are actively applying for jobs in organizations where part-time work is available. This is not a direct effect of part-time work as such, but an effect of systematic differences between employees employed in full-time versus part-time jobs. Studies have indicated that people with health problems apply for positions in organizations where they expect job pressure to be lower [1]. As job pressure probably will be lower the less hours one is working, one could also expect potential employees with health problems to seek out organizations with part-time jobs. This again will increase the probability of absenteeism as bad health is unequivocally linked to higher absenteeism.

A variant of the selection/attraction mechanism is that part-time jobs are mainly occupied by females, mainly married women and women with children [69]. This is probably linked to work-family conflict/balance as women still are the ones primarily taking responsibility for home and children. Furthermore, there is solid evidence that sickness absence is higher for women than for men [70], both across sectors [1, 21], occupations, and nations [23]. Therefore, one should expect organizations with many part-time jobs to employ more women, and thus to have higher absence rates.

A final variant of the selection/attraction mechanism is that part-time jobs seem to be occupied by employees with lower education than what one finds in full-time jobs [71]. Several studies indicate that lower levels of education are related to higher levels of absenteeism [12, 72, 73]. Again, we will assume that organization with high levels of part-time jobs will attract more employees with lower levels of education, resulting in higher levels of absenteeism.

Thus, we propose an alternative hypothesis:

H1alt: Absence at the organizational level will increase with higher levels of part-time work in the organization.

## Methods

### Data

Units in the study are Norwegian municipalities. They are multifunctional organizations delivering public services such as basic primary and secondary education (6 to 16 years), primary health care including institutional care for elderly, childcare and kindergartens, as well as services connected to culture (libraries, youth clubs, etc.), emergency (fire services, emergency care), and technical services like road and park maintenance. All Norwegian municipalities provide similar services and thus share the same occupational profile. Still, they vary extensively both in absenteeism (between 4.3 and 12.6% in 2019) and the use of part-time work (mean position percent between 60.5 and 93.7 in 2019). Norwegian municipalities thus represent an excellent opportunity to compare organizational entities with similar occupational composition, but with different use of part-time work.

Data cover the years 2014 to 2019. In 2020, a wave of municipal amalgamations was conducted, reducing the number from 428 to 356. As radical organizational change affects sickness absence [74], we decided to not include municipalities after 2019. Municipalities amalgamated in the period between 2014 and 2019 have also been removed from the analysis. The capital – Oslo – is both a municipality and a county has also been removed. The total number of organizations in the current analysis is 422, constituting a panel of potentially 2532 observations. As a few municipalities miss information on some of the confounding variables (see next paragraph) and have thus been excluded, the actual number of units/observations is slightly lower (Obs = 2503) in the subsequent bivariate and multivariate analyses.

### Variables

Absenteeism is empirically defined as sickness absence or the “(…) absence from work due to ill health” [34]. As most countries have arrangements allowing employees to stay away from work based on their own judgement, this definition opens for both doctor certified and self-reported illness, again blurring the line between “real” sickness absence and truancy.

Objective data on sickness absence and part-time/full-time work was retrieved from the National association of local and regional authorities. Municipalities are by law obliged to register data concerning wages, titles, absence, etc., and to report it to the central register every year. The register represents an objective oversight, thus avoiding common problems associated with self-reporting [75]. Absence is defined as number of days during a year [76, 77]. Only employees with a registered engagement are included, and any employees below 18 years of age and apprentices are excluded. Employees in temporary (short time) employment, employed on an hourly basis, and those employed less than 12 months in the actual year are also excluded. Absence is registered at the individual level and aggregated to the organizational (municipal) level as the “sickness absence percent”. The formula for computing this is:

(days of sickness absence/planned workdays) * 100

The term “planned workdays” takes into consideration whether one is working full-time or part-time. For a “normal” employee the number of planned workdays for a year is 235, with some variation depending on occupations (teachers for instance, have 196 days per year) and whether one is working shift work (those working shift work including night shifts have a lower number of planned workdays). An employee working 50% part-time will thus have a planned number of 117.5 workdays per year. The number reported here is thus the number of workdays lost to sickness absence during a year for the whole municipality, in relation to the number of workdays that were planned for the whole municipality.

Part-time work is computed as “mean position percent”, and is the ratio between the number of employees in the organization and the number of planned or “normal” full-time positions:

(planned full-time positions/number of employees) * 100

Planned full-time positions are estimated on hours expected work during a year, again differing somewhat between occupations and shiftwork or not. Part-time/full-time work is thus operationalized as a variable ranging theoretically between zero and 100. This gives us the opportunity not only to scrutinize eventual differences between part-time and full-time workers, but also to see any gradual effects and differences between different degrees of part-time work and to estimate eventual non-linear effects [78, 79].

### Confounders

We also control for possible confounders, i.e., phenomena affecting both the cause (part-time work) and effect (absenteeism) simultaneously [80]. First, we control for demographic composition of the organization, especially its gender and educational composition as previous research has indicated that these variables show consistent correlation with both absenteeism and part-time work. Second, we control for organizational size. Larger organizations may have better possibilities to “fill up” positions, and thus to offer full-time jobs. This will probably be more difficult for smaller organizations, having problems – i.e., economic resources – to offer full-time jobs to all employees. Earlier empirical studies also indicate that size is negatively related to organizational commitment [64, 81] and social control and that sickness absence seems to increase with increasing organization size [82]. The research on the relationship between organizational size and absenteeism are, however, scarce [83]. Data on both these variables are collected from the municipal register where size is operationalized as number of estimated full-time positions and gender composition as the number of estimated full-time positions for employed women in relations to all estimated full-time positions.

We also include indicators on the general economic situation of the organization. Organizations experiencing fiscal stress may be forced to exploit the workforce more optimally than organizations with economic slack, and part-time work may be an indicator of cost savings rather than offering flexible work [62]. Thus, organizations under fiscal stress will probably calculate workforce cost more exactly, offering positions with more diverse working hours to exploit the labor most efficiently. Moreover, fiscal stress will also most likely mean less resources available for employees. Following job-demand and resources theory [13, 84], lack of resources will often result in burn-out, something that again may end up in sickness absence [85]. Fiscal stress is defined in two different ways. First, free income per capita measured in Norwegian kroner (NOK) is an estimation of how much “there is left” for use in the municipality after the basic requirements following national laws have been covered. Second, a yearly net surplus is a measure of how much resources a municipality has for investments/funds after running expenses have been covered. Both are measures of the available resources in the municipality.

Furthermore, we consider important aspects of the organization’s environment. First, as noted by several economists, is the notion that absenteeism is correlated with availability of jobs outside the organization, i.e., how tight the labor market is [24, 67]. In a tight labor market, that is where it is easy for an employee to find alternative employment and difficult for the employer to hire substitutes for their employees, incentives to go to work will be less intense. The employee knows that it is not likely to get fired for being away from work, and if one does, finding alternative work may be rather easy. To attract personnel that usually would not consider taking a job, organizations may find it rational to offer jobs that are not full-time. Offering such jobs may attract people that would like to work, but not to have a full job. A tight labor market may thus result in a higher differentiation of jobs, including the number of working hours defined. Labor market “tightness” is measured by the percentage of registered unemployed person in relation to all persons in normal working age (18–67) in the municipality. The higher this number, the higher the unemployment rate in the municipality.

Second, we noted above that part-time might be a way to ease conflicts between work and family, leading especially women to work part-time instead of full-time. This conflict can be relieved through offering kindergartens and other home help services, either through public or private providers. A good coverage of such services may result in more people taking full-time jobs and reduce the necessity to take some days of to care for children. Studies indicate that childcare load is a significant contributor to increased absenteeism [86]. Providing services that may give some relief from this strain may thus reduce days away from work. The provision of child support for employees is measured by the number of children in the municipality having a registered place in a kindergarten in relation to the number of children in the age from 1 to 5 years in the municipality. The higher this number/rate, the better provision of kindergartens in the organization’s environment. There is no official statistic on educational level of municipal employees. As municipalities will to large degree be dependent on a local labor market, we include the percentage of the population in the municipality having the lowest educational level (basic school 1–10).

Data on confounders containing information on the municipal situation were all retrieved from the Norwegian National Bureau of Statistics (SSB).

### Statistical methods

The first step was to inspect the distributions of the dependent and independent variables. Both sickness absence and percentage position are normally distributed both pooled and for individual years (Kolmogorov- Smirnov-tests p > .10). Of the other independent variables, only the number of full-time employees was highly positively skewed because many Norwegian municipalities are small, and that the number of employees almost linearly follow the number of inhabitants. This variable is log-transformed for all years. Next, we computed bivariate correlations between all variables in the analysis using Pearson’s r. The significance of these correlations was tested using a two-tailed chi-square test. None of the independent variables are so highly correlated as to fear collinearity. Distributions and correlations are displayed in Table 1.

To fit the model for estimating the effect of part-time work on sickness absence we first compared a model with municipality fixed effects with a random effects model. A fixed effect model is primarily aimed at estimating changes that occur over time while controlling for time invariant differences between municipalities, thus controlling for non-observed differences between units and reducing the danger of model misspecification due to omitted variables. However, it also assumes that the main independent variable – here: percentage employment - change significantly over time. To test whether a random effect model was preferable to the fixed model, we conducted a Hausman test. This was significant (p < .001), leading us to reject the null hypothesis that a random effect model was preferrable. Next, we conducted the LM test (Breusch-Pagan Lagrange multiplier) to see whether an ordinary least square (OLS) model would fit the data better than the random effects model. Again, the test was highly significant (p < .001), indicating that a random effects model is preferable to an OLS model. We also tested whether time fixed effects would provide a better fit to the data than a model without such effects by conducting a Wald test on whether the coefficients for each year are jointly equal to zero. The test was highly significant (p < .001), indicating that time fixed effects should be included in the model. Using a modified Wald statistic test for groupwise heteroskedasticity yielded a highly significant result (p < .001), leading us to choose a model with robust standard errors to counter this problem. To highlight the effect of the focus variable – part-time work – we conducted the regression analysis in two steps. First, we introduce all the possible confounders as independent variables and then we include part-time work.

## Results

Table 1 display descriptive statistics for the pooled data (all years) included in the analysis, as well as the bivariate correlations between them.

**Table 1:** Variables in the analysis, pooled data 2014–2019. Descriptive statistics (means, standard deviation) and correlations (Pearson’s r). N = 2503

Name	Mean(std)	Y	X1	X2	X3	X4	X5	X6	X7
Y Sickness absence (%)	9.6 (1.5)	1							
X1% position	0.76 (0.04)	0.19***	1						
X2 Organization size (log)	787.8 (2081.8)	0.17***	0.32***	1					
X3 Ratio female employees	0.79 (0.04)	− 0.02	− 0.18***	− 0.09***	1				
X4 Free income per capita	61,674 (12,484)	− 0.13***	− 0.11***	− 0.70***	− 0.17***	1			
X5 Net yearly surplus (% of running costs)	2.6 (2.2)	− 0.07***	0.03	− 0.02	− 0.05**	0.07***	1		
X6 Ratio children in kindergarten	0.76 (0.32)	− 0.02	− 0.10**	− 0.04*	− 0.07**	0.01	− 0.03	1	
X7 Ratio secondary education	0.36 (0.08)	− 0.14***	− 0.25***	− 0.22***	0.03*	0.02	− 0.01	0.03	1
X8 Unemployment rate (%)	2.2 (0.8)	0.21***	0.12***	0.15***	− 0.08***	− 0.12***	− 0.01	− 0.05**	− 0.15***

* p < .10, ** p < .05, *** p < .01

Over the whole period the mean sickness absence in Norwegian municipalities was 9.6%, with a 95% confidence interval (CI) between 9.5 and 9.7, while the mean percentage position was 76% (CI 75–77%). The initial correlation analysis indicates that the higher the mean percentage position, the higher the sickness absence. This gives initial support for our alternative hypothesis. The correlation analysis also gives preliminary support for the assumptions that there would be lower absenteeism in municipalities with good economy and with a high proportion of highly educated employes, and higher absenteeism in larger municipalities. Contrary to our initial assumptions, higher rate of unemployment is associated with higher absenteeism, while kindergarten coverage seems to be unrelated to absenteeism. However, several of the explanatory variables are intercorrelated, necessitating a multivariate analysis to isolate the statistical effects of individual variables. Table 2 displays the result from two regression equations with fixed municipality and time effects.

**Table 2:** Linear fixed effects (municipality, year) regression. Unstandardized coefficients. Robust standard errors in (). Dependent variable = sickness absence (percent). Groups = 418, Observations = 2503. * = p < .10, ** = p < .05, *** = p < .01

Variable	Model 1	t-value	Model 2	t-value
Percent position			1.56 (1.56)	0.99
Organization size (log)	0.94 (0.68)	1.38	0.92 (0.67)	1.37
Ratio female employees	2.78* (1.64)	1.69	2.87** (1.63)	1.75
Free income per capita (1000NOK)	0.044*** (0.02)	2.11	0.044*** (0.014)	2.12
Net yearly surplus (percent)	− 0.008 (0.01)	− 0.73	− 0.008 (0.009)	− 0.69
Ratio children in kindergarten	1.18** (0.59)	1.99	1.16** (0.46)	1.97
Ratio secondary education	1.78 (2.06)	0.86	1.68 (2.44)	0.82
Unemployment rate (%)	0.002 (0.05)	0.05	0.005 (0.05)	0.09
F-value	1.8**		1.8**	
Overall R-squared	0.01		0.02	
Rho (icc)	0.62		0.61	

The use of part-time work in the municipalities, has a positive, but non-significant effect on sickness absence measured as percent of expected working days in a year. Thus, the results support none of the proposed hypotheses. Part-time work seems to be unrelated to absenteeism. The effect of percent position is positive indicating increased absenteeism with more full-time work, but the confidence interval overlaps zero. Only one of the initial assumptions, that a higher proportion of female employees is associated with higher absenteeism, is supported. Two effects are opposite to our assumptions: absenteeism is higher in municipalities with a good economy and high kindergarten coverage. Overall, only a small fraction of the variation in absenteeism is explained by the variables in this analysis.

Even though earlier tests indicated that the choice of model used is the best, we compared the results in Table 2 with outcomes for the focus variable when running random effects model and the OLS model. Both these models yielded significant and positive effects of the focus variable percent position (OLS coefficient = 4.90, p < .001, random effects model coefficient = 3.07, p < .001), showing the importance of controlling for unobserved differences between units/municipalities. As the dependent variable is bounded between 0 and 100, we also conducted a fractional regression analysis. This yielded results almost identical to the OLS regression. The high rho shows the importance of differences between municipalities. More than 60% of the variation in sickness absence at the municipal level is explained by (unobserved) differences between municipalities.

## Discussion

The use of part-time work in the public sector is controversial, and much debated due to several negative effects for those working less than “normal”. Part-time work is, for instance, linked to lower wages, lower job security and fewer training opportunities [27]. Due to these negative effects, the Norwegian parliament changed the general law on the “working environment”, introducing the following paragraph in Sect. 14-1b: “As a rule, an employee shall be employed full-time”^1^. Still, part-time work is common and probably increasing, especially in public organizations providing health and social services [26].

As shown in the theory section, questions have also been posed whether part-time work is associated with higher or lower absenteeism. This study shows that part-time work is not associated with absenteeism at the organizational or aggregated level. In short, organizations employing many part-time workers do not experience higher absence rates than organizations employing few part-time works. Thus, the *net organizational effect* of part-time work on sickness absence is neither positive nor negative.

This is important information for those responsible for designing the “human resource architecture” of an organization [33] where the use of part-time work is an important component. From the organization point of view, organizing work as part-time has many benefits. It increases numerical flexibility as part-time workers can more easily expand their working capacity on short notice than full-time workers, and they can function as a “emergency reserve” to call upon when employees suddenly “go missing” (accidents, sickness) [87, 88]. They may also attract highly sought-after personnel through offering flexible work hours, and they may make optimal use of the workforce easier by avoiding slack many times inherent in full-time positions [89]. For many organizations relying on 24/7 production, part-time work may be necessary to be able to fill complex rotation arrangements during the day and week [56].

Given these beneficial *organizational* effects of part-time work, it is essential also to gain knowledge on possible negative organizational consequences or costs of employing people in part-time positions. One field where our knowledge is limited is whether organizations’ use of part-time work affects absenteeism. This study suggests that the use of part-time work is unrelated to absenteeism, supporting “zero-findings” from other studies [39–41].

Reflecting on this finding, it is important to have in mind that this conclusion only holds at the organizational level. Drawing the inference that part-time work is unrelated to sickness absence at the individual level would be an ecological fallacy. As discussed in the theory section of this article, part-time work may have both positive and negative effects on absenteeism concerning individuals, depending on individual characteristics and situational factors. For some, part-time work may be a way to ease work-family tensions, while others may find it stressful because they want to work more hours to get a higher income. For individuals, the effect of part-time work on absenteeism will probably depend on a multitude of contingency factors like personal characteristics (gender, age, education, personality), family situation (children at home, single or not, parent’s age, etc.), and expectations from peers, local community, and society at large [33]. At the aggregate – or organizational – level, however, these positive and negative effects may balance each other out, as the conclusions in this study indicate.

Although this study has several strong aspects – objective data both on dependent and independent variable, panel data making it possible to study the phenomenon over time and controlling for unobserved and potentially important explanatory factors, studying large and multifunctional organizations covering a wide range of occupations and professions – it also must be interpreted in its context. First, it is possible that studies of different services within each municipality would yield different results. The effect of part-time work on absenteeism may for instance differ between those working in the general administration and those working in primary schools.

It is also an open question whether the findings in this study within the Norwegian context will travel to other contexts. It is quite likely that the findings will travel to the same organizations in similar countries (northern Europe) with much of the same responsibilities and tasks delegated to the local level. It is probably more questionable if the finding also applies also to other public organizations, and finally to private business organizations. As these contexts differ widely, the effects – or “no-effects” – detected in this study may change, even dramatically [90].

Even though this study indicates that the level of part-time work in organizations is unrelated to sickness absence, one should not jump to the conclusion that this can be used as a one-sided argument for using part-time as an overall organizational strategy (Human Resource Management (HRM) policy). As noted by Kwon & van Jaarsveld [62], it seems of utter importance whether the organizational motivation for extensive use of part-time work is based on flexibility or cost-savings. If it is the latter, one should expect part-time employees to be less integrated into the organization and have higher levels of absenteeism. If part-time is an element in a policy to increase individual flexibility, one may assume that this is done based on an individual evaluation of the fit between the person’s wishes and needs and working hours. This will in turn be a strong indicator on the organization’s willingness to integrate the individual needs into the organizational policy, again probably increasing commitment and attachment, and lowering absenteeism. Further studies on the links between part-time use and absenteeism should include measures on the organization’s motivation.

Furthermore, there should be more studies on the links between organizational characteristics and absenteeism at the organizational level. Transforming knowledge about how different variables are connected to absenteeism at the individual level to knowledge on how to design organizational policies is in many instances difficult. Therefore, research on how organizational characteristics like the extension of part-time work, shift-work and other work arrangements are linked to absenteeism at the organizational level might produce very useful information on how to design policies to meet some of the greatest challenges for all organizations: absenteeism.

## Data Availability

All data was retrieved from publicly available databases. Data on sickness absence was collected from The Norwegian Association of Local and Regional Authorities (https://www.ks.no/fagomrader/statistikk-og-analyse/fravar/). Data on confounders was retrieved from Statistics Norway (https://www.ssb.no/en/offentlig-sektor/kostra). The combined dataset used in the current study can be made available from the corresponding author on reasonable request.

## Footnotes

1 https://lovdata.no/dokument/NLE/lov/2005-06-17-62/*#KAPITTEL_15.
